# Supplementary material for: Modulation of Gut Microbiota by Cacao: Insights from an In Vitro Model
Source: Curr Issues Mol Biol. 2025 Jun 3;47(6):414. doi: 10.3390/cimb47060414 (PMC12191874; doi:10.3390/cimb47060414)
Supplement: Supplementary file 1 [file cimb-47-00414-s001.zip › Supplementary information_Figures_rev2.pdf]

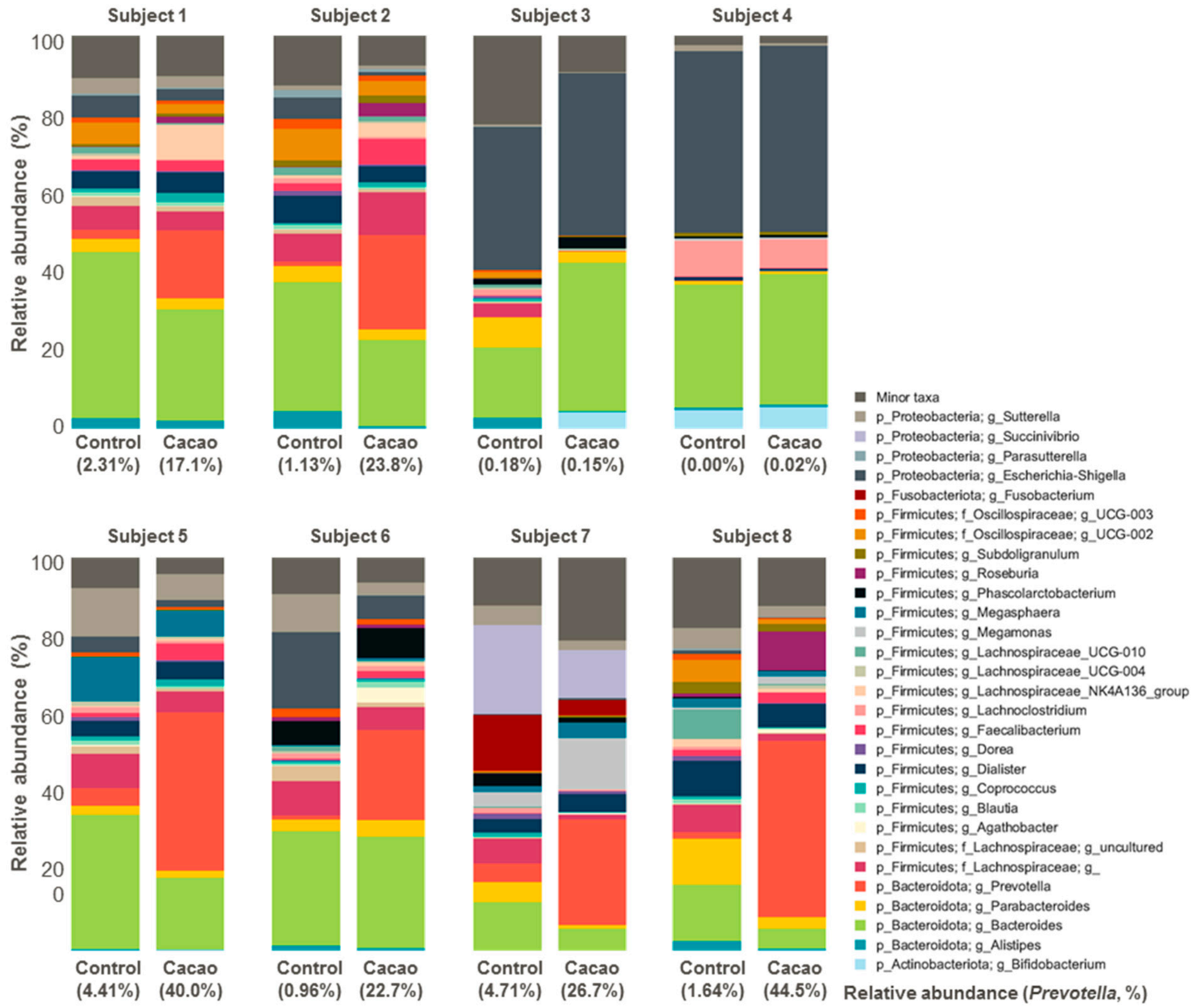

Figure S1. Bacterial taxonomy at the levels of phylum and genus (top 30 bacterial taxonomies) of 8 subjects belonging to the *Prevotella* type. Control, non-treated control group; Cacao, cacao-treated group.

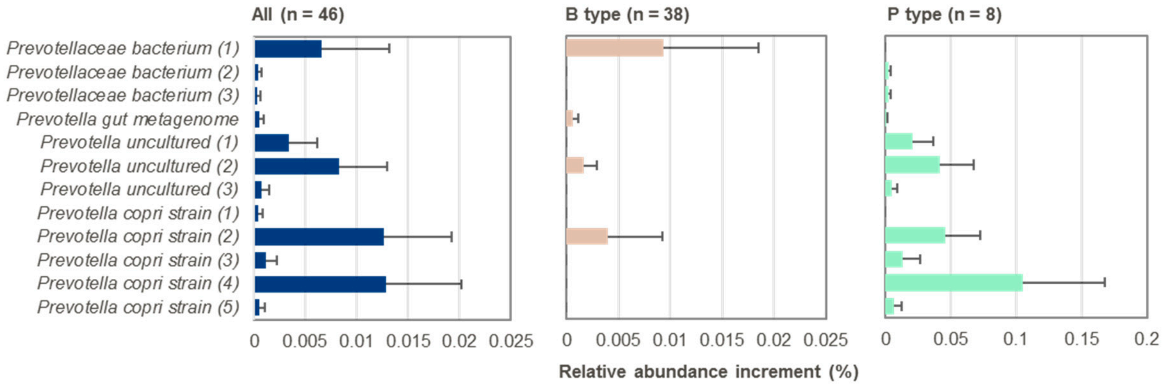

Figure S2. Relative abundance increment of *Prevotella* in the cacao group compared to the control group.

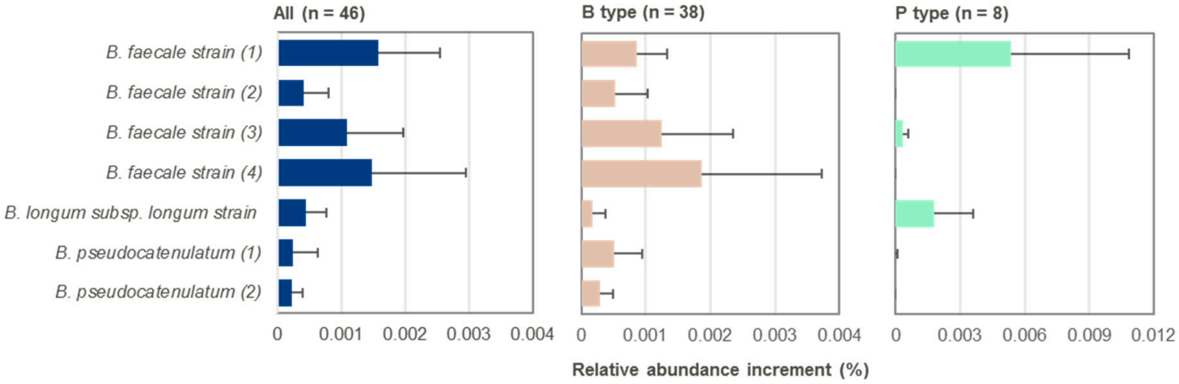

Figure S3. Relative abundance increment of *Bifidobacterium* in the cacao group compared to the control group.
